# KIOM-79 Prevents Lens Epithelial Cell Apoptosis and Lens Opacification in Zucker Diabetic Fatty Rats

**DOI:** 10.1155/2011/717921

**Published:** 2010-09-07

**Authors:** Junghyun Kim, Chan-Sik Kim, Eunjin Sohn, Hyojun Kim, Il-Ha Jeong, Jin Sook Kim

**Affiliations:** Diabetic Complications Research Center, Division of Traditional Korean Medicine (TKM) Integrated Research, Korea Institute of Oriental Medicine (KIOM), 483 Exporo, Yuseong-gu, Daejeon 305-811, Republic of Korea

## Abstract

Damage of lens epithelial cells (LECs) has been implicated in cataract formation. The aim of this study was to investigate the protective effect of KIOM-79, a combination of four plant extracts, on LECs. We examined the levels of advanced glycation end products (AGEs), nuclear factor-kappaB (NF-*κ*B) activation and inducible nitric oxide synthase (iNOS) expression in LECs during cataract development using the Zucker diabetic fatty (ZDF) rat, an animal model of type 2 diabetes. KIOM-79 was orally administered by gavage to ZDF rats once a day for 13 weeks. Apoptosis was detected by TUNEL assay, and NF-*κ*B activation and iNOS expression were studied by southwestern histochemistry and immunohistochemistry, respectively. In diabetic cataractous lenses, TUNEL-positive LECs were markedly increased 20-fold, and AGEs were highly accumulated (2.7-fold) in LECs. In addition, both NF-*κ*B activation, and iNOS expression were significantly enhanced 3- to 5-fold, respectively, compared to levels found in normal ZL rats. However, the administration of KIOM-79 delayed the development of diabetic cataracts and prevented LEC apoptosis (70%) through the inhibition of AGEs, NF-*κ*B-activation and iNOS expression. These observations suggest that KIOM-79 is useful in inhibiting diabetic cataractogenesis and acts through an antiapoptotic mechanism to protect LECs from injury.

## 1. Introduction

Cataract, characterized by cloudiness or opacification of the eye lens, is the leading cause of blindness worldwide. Although the pathogenesis of diabetic cataract is not known, various biochemical pathways, such as the polyol pathway, the generation of advanced glycation end products (AGEs) and oxidative stress, have been implicated [1]. Recently, the damage of lens epithelial cells (LECs), induced by chronic hyperglycemia, is considered to be important in perturbing lens homeostasis [2]. The eye lens contains only a single layer of epithelial cells in its anterior surface. LECs are thought to protect underlying fibers from injury [3] and maintain the transparency of the lens [4]. Thus, the damage of this cell might be linked to cataractogenesis.

AGEs are sugar-derived, irreversible protein modifications that have been implicated in the pathogenesis of diabetic complications [5]. It was reported that AGEs enhance apoptosis of retinal pericytes, corneal endothelial cells, neuronal cells, and renal mesangial cells through increasing oxidative stress or via induced expression of pro-apoptotic cytokines [6–8]. Furthermore, AGEs induce a dose-dependent activation of nuclear factor-kappaB (NF-*κ*B) in LECs *in vitro* [9]. The activation of NF-*κ*B might play an important role in modulating the function of LECs [10, 11]. NF-*κ*B activation due to ultraviolet irradiation induces apoptosis in LECs [11]. NF-*κ*B activation in ocular tissue also induces both the loss of retinal pericytes [12] and retinal capillary cell death [13]. Activated NF-*κ*B binds with target DNA in cellular nuclei, which activates the gene for induction of cellular death.

Nitric oxide (NO) is a signaling molecule that mediates a variety of physiological processes, including neurotransmission, vasodilation, and host cell defense. The expression of inducible nitric oxide synthase (iNOS) is induced by cytokines, AGEs, and NF-*κ*B [14, 15], and its induction results in the release of excessive amounts of NO [16, 17]. In several ocular diseases, including uveitis, retinitis, glaucoma, and cataract, the alteration of iNOS expression has been reported [18–21]. In addition, *in vitro* cataract formation, generated by high levels of glucose, is inhibited by a nitric oxide synthase inhibitor [22]. Therefore, these findings suggest that the inhibition of AGEs, NF-*κ*B activation, and iNOS expression represent potential therapies for the prevention of LEC injury and diabetic cataractogenesis. 

 KIOM-79 is a mixture of the 80% ethanol extract of parched Puerariae radix, gingered Magnoliae cortex, Glycyrrhizae radix, and Euphorbiae radix. These herbs are used in traditional Korean medicine for a variety of medical purposes, including diabetes [23]. Our previous work showed that KIOM-79 prevented the development of diabetic nephropathy in streptozotocin-(STZ-) induced type 1 diabetic rats [24] and nonobese type 2 diabetic Goto-Kakizaki rats [25]. In addition, previous studies have reported that KIOM-79 treatment shows a strong inhibitory effect on AGE formation *in vitro*  [24] and inhibits STZ-induced apoptosis of the pancreatic betacells [26]. Recently, KIOM-79 was shown to prevent apoptosis of retinal ganglion cells in diabetic db/db mice without affecting blood glucose [27]. Despite the various effects of KIOM-79 on diabetic complications, the effect on LEC injury and diabetic cataract is limited. Therefore, in this study, we evaluated the effect of KIOM-79 treatment on LEC injury and cataractogenesis in the Zucker diabetic fatty (ZDF) rat, an animal model of type 2 diabetes. Based on the above-mentioned therapeutic targets, we specifically evaluated the ability of KIOM-79 to inhibit AGE accumulation, NF-*κ*B activation and iNOS expression in LECs and identified an anti-apoptotic property of KIOM-79.

## 2. Materials and Methods

### 2.1. Preparation of KIOM-79

KIOM-79 was prepared as previously described in [23, 27]. Briefly, the cortex of *Magnolia officinalis*, radix of *Pueraria lobata*, radix of *Glycyrrhiza uralensis,* and radix of *Euphorbia pekinensis *were collected from plants obtained from Gamsuk Province (China). Magnoliae cortex (100 g) was simmered with 3 g of Zingiberis rhizoma for 60 minutes. Puerariae radix (100 g) was stir-roasted at 75°C for 45 minutes. Equal amounts of gingered Magnoliae cortex, parched Puerariae radix, Glycyrrhizae radix, and Euphoriae radix were mixed, pulverized, extracted in 80% ethanol for one week at room temperature, concentrated with a rotary evaporator, and lyophilized. The entire procedure was repeated four times.

### 2.2. Standardization of KIOM-79

Identification of the major compounds in KIOM-79 was determined by high performance liquid chromatography (HPLC, Agilent 1200 HPLC system, Agilent, CA, USA). A Spherex C-18 analytical column (250 × 4.6 mm, 5.0 *μ*m, Phenomenex, CA, USA) was used with the mobile phase consisted of acetonitrile (A) and 0.1% acetic acid in water (B). The mobile phase gradient elution was programmed as follows: 95–70% B (0–30 minutes), 70–40% B (30–40 minutes), 40–0% B (40-45 minutes), and 100% A (45–50 minutes). The column temperature was maintained at 30°C, flow rate set at 1 ml/minutes, and sample injection volume was set at 10 *μ*l.

### 2.3. Animals and Experimental Design

Male 6-week-old ZDF rats (ZDF/Gmi-fa/fa) and Zucker lean (ZL) counterparts (ZDF/Gmi-lean) were purchased from Charles River Laboratory (Waltham, MA, USA) and acclimated for 1 week prior to the study. Rats were individually housed in plastic cages and maintained at 24°C ± 2°C with a 12-hour light : dark cycle and received a diet of Purina 5008 (Ralston Purina, St. Louis, MO, USA) and tap water *ad libitum*. Rats were divided into 3 groups of 8 rats, according to their initial blood glucose concentration as follows: (1) normal ZL rats, (2) vehicle-treated ZDF rats and (3) ZDF rats treated with KIOM-79 (50 mg/kg body weight). The dosage of freeze-dried powder was calculated based on the minimum human equivalent dosage of raw herbs. KIOM-79 was dissolved in *distilled* water and administered daily by oral gavage for 13 weeks. In normal ZL rats and vehicle-treated ZDF rats, water was given orally for 13 weeks. Blood glucose levels and body weights were monitored consecutively, and glycated hemoglobin was determined by a commercial kit (Unimate HbA1c, Roche Diagnostics, Mannheim, Germany). All procedures involving rats were approved by the Korea Institute of Oriental Medicine Institutional Animal Care and Use Committee.

### 2.4. Analysis of Cataract Development

Following thirteen weeks of treatment, eyes were enucleated under deep anesthesia, following an intraperitoneal injection of pentobarbital sodium (30 mg/kg body weight). The lenses were excised from eyeballs under an optical microscope and transferred onto 6-well plates each containing 5 ml of a saline solution. Lens opacity was then measured under an optical microscope. Score of lens opacity is determined as follows according to the classification of Ao et al. [28]: 0, clear normal lens; 1, peripheral vesicles; 2, peripheral vesicles and cortical opacities; 3, diffuse central opacities; 4, matured nuclear cataract.

### 2.5. Apoptosis Assay

TUNEL assays were performed with the DeadEnd apoptosis detection system (Promega, Madison, WI, USA), according to the manufacturer's instructions. Apoptotic cells were detected with peroxidase-conjugated streptavidin in the lens section. For quantitative analysis, TUNEL-positive nuclei were then counted.

### 2.6. Immunohistochemical Staining

Immunohistochemistry was performed as previously described in [29]. Antibodies were mouse anti-AGEs (6D12, Cosmo bio, Tokyo, Japan) and rabbit anti-iNOS (Cell Signaling, MA, USA). The 6D12 antibody recognizes both N^*ε*^-(carboxymethyl) lysine-(CML-) and N^*ε*^-(carboxyethyl) lysine-(CEL-) protein adduct as epitopes. For detection of AGEs and iNOS, the sections were incubated with the LSAB kit (DAKO, CA, USA) and visualized by 3,3′-diaminobenzidine tetrahydrochloride. For morphometric analysis, the immunoreactive intensity per unit area (0.32 mm^2^) in a total of 5 fields was determined using Image J software (NIH).

### 2.7. Immunofluorescence Staining

Immunofluorescence staining was performed on the lens sections. The lens sections were incubated with rabbit anticleaved caspase-3 antibody (Cell Signaling, MA, USA). The sections were then incubated with tetramethyl rhodamine isocyanate-(TRITC-) conjugated goat antirabbit IgG (Santa Cruz, CA, USA). The intensity of the fluorescence was analyzed in five randomly selected mm^2^ areas using Image J software (NIH).

### 2.8. Western Blotting Analysis

Whole lenses from each group were homogenized in RIPA buffer (20 mM Tris-HCl pH 7.4, 1.0% Triton X-100, 150 mM NaCl, 100 *μ*M leupeptin, 100 *μ*M aprotinin, 1 mM EDTA, and 1 mM EGTA). The insoluble materials were removed by centrifugation. Proteins were then separated by SDS–polyacrylamide gel electrophoresis and transferred to nitrocellulose membranes (Biorad, CA, USA). Membranes were probed with anti-AGEs antibody (Cosmo bio), and then the immune complexes were visualized with an enhanced chemiluminescence detection system (ECL; Amersham Bioscience, NJ, USA). Protein expression levels were determined by analyzing the signals captured on the nitrocellulose membranes using an image analyzer (Las-3000, Fuji photo, Tokyo, Japan).

### 2.9. Southwestern Histochemistry for the Detection of Activated NF-*κ*B

To localize the NF-*κ*B activity in lens epithelial cells, *in situ* southwestern histochemistry was performed as described by Hernandez-Presa et al. [30]. Briefly, complementary oligonucleotides containing the NF-*κ*B binding consensus sequence were synthesized as follows: 5′-AGTTGAGGGGACTTTCCCAGGC-3′. The probe was labeled with digoxigenin (DIG oligonucleotide 3′-end labeling kit, Roche Diagnostics, Mannheim, Germany). Formalin-fixed, paraffin-embedded lens sections were dewaxed, rehydrated, and incubated with 5 mM levamisole (Dako) to inhibit endogenous alkaline phosphatase, and fixed with 0.2% paraformaldehyde for 30 minutes at 28°C. Sections were subsequently digested with pepsin A (433 U/mg; Sigma), washed twice with buffer 1 (10 mM HEPES, 40 mM NaCl, 10 mM MgCl_2_, 1 mM DTT, 1 mM EDTA, 0.25% BSA, pH 7.4), then with 0.1 mg/ml DNAse I, and finally washed once with buffer 2 (10 mM HEPES, 40 mM NaCl, 1 mM DTT, 10 mM EDTA, 0.25% BSA, pH 7.4) to stop the reaction. The labeled probe (100 pM) diluted in buffer 1 containing 0.5 mg/ml poly (dI-dC) (Pharmacia LKB, Piscataway, NJ, USA) was applied overnight at 37°C. After washing, sections were incubated for 1 hour in blocking solution (0.01X SSC, 0.01% SDS, 0.03% Tween 20, 0.1 M maleic acid, 0.15 M NaCl, pH 7.5) and with antidigoxigenin antibody conjugated with alkaline phosphatase (1 : 250 in blocking solution; Roche Diagnostics) 1 hour at 37°C. The color reaction was developed using nitroblue tetrazolium (NBT, Roche Diagnostics) and 5-bromo-4-chloro-3-indolylphosphate (BCIP, Roche Diagnostics). The number of cells positive for NF-*κ*B activation was then counted with computer-assisted image J software (NIH). As negative controls, the following were used: (1) absence of probe, (2) mutant NF-*κ*B probe labeled with digoxigenin, and (3) competition assays with a 200-fold excess of unlabeled NF-*κ*B followed by incubation with labeled probe.

### 2.10. Statistical Analysis

Statistical evaluation of the results was performed using a one-way analysis of variance (ANOVA) followed by Tukey's multiple comparison test using GraphPad Prism 4.0 software (Graph pad, CA, USA).

## 3. Results

### 3.1. HPLC Analysis of KIOM-79

The major components of KIOM-79 include puerarin, glycyrrhizin, honokiol, and magnolol. The amounts of puerarin, glycyrrhizin, honokiol, and magnolol in KIOM-79 were 4.82, 2.17, 0.87, and 1.69%, respectively (Table 1).

### 3.2. Body Weight, Blood Glucose, and HbA1c Levels

At 21 weeks of age, all ZDF rats developed hyperglycemia in contrast to the normal ZL rats. As shown in Table 2, the untreated ZDF rats had a more than fourfold increase of fasting blood glucose levels and increased HbA1c levels. Body weights of the vehicle-treated ZDF rats were elevated approximately 80% compared to the normal ZL rats. KIOM-79 induced a minor decrease of blood glucose levels but did not affect HbA1c levels or body weight.

### 3.3. Cataract Formation

**Figure 1:**
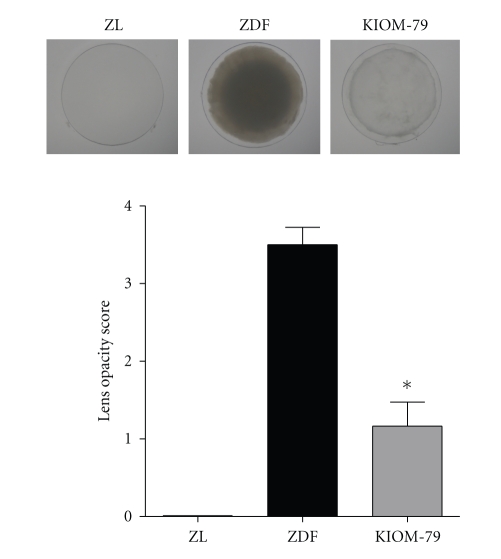
Analysis of lens opacity. Representative images of lenses in each group. Lens opacity was analyzed (score 0 to 4) from each lens from a normal Zucker lean rat (ZL), Zucker diabetic fatty rat (ZDF), and ZDF rat treated with KIOM-79 (KIOM-79). All data are expressed as means ± SE, *n* = 8. *P < .01 versus vehicle-treated ZDF rats.

At the end of the thirteen-week study, the lens opacity score of vehicle-treated ZDF rat lenses was more than 3 (Figure 1). In normal ZL rats, no lenses displayed cataract formation (score 0). However, the administration of KIOM-79 to ZDF rats decreased the score of lens opacity to 1.16. This result indicated that KIOM-79 reduced the development of cataracts in ZDF rats.

### 3.4. Apoptosis of LECs

**Figure 2:**
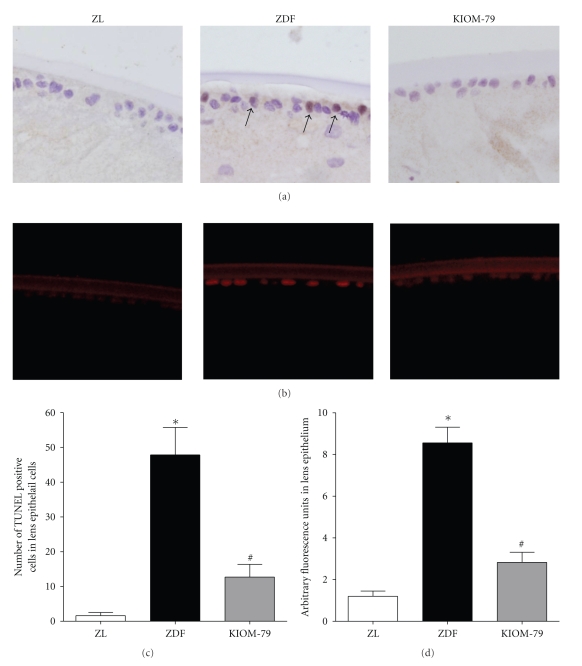
Apoptosis of LECs. (a) The lens sections from a normal Zucker lean rat (ZL), vehicle-treated ZDF rat (ZDF), and ZDF rat treated with KIOM-79 (KIOM-79) were stained with a TUNEL kit. Apoptotic LECs (arrow) were observed in vehicle-treated ZDF rats. (b) Immunofluorescence staining of cleaved caspase-3. Immunofluorescence signals for cleaved caspase-3 (red) were mainly detected in the lens epithelium of ZDF rats. X400 magnification. (c and d) Quantitative analysis of TUNEL-positive and cleaved caspase-3-positive cells in LECs. All data are expressed as means ± SE, *n* = 8. *P < .01 versus normal ZL rats, ^#^ *P* < .01 versus vehicle-treated ZDF rats.

In TUNEL assays, the lens sections from vehicle-treated ZDF rats showed many TUNEL-positive cells in the anterior epithelial and equatorial regions (Figure 2(a)). In immunofluorescence staining, the expression of cleaved caspase-3 protein was significantly increased 7.0-fold higher than normal ZL rat expression patterns (Figures 2(b) and 2(d)). This result suggests that a portion of the LECs underwent apoptosis under diabetic conditions. This enhanced apoptotic cell death in ZDF rats was significantly suppressed, by 71%, with KIOM-79 treatment (Figure 2(c)).

### 3.5. AGE Accumulation in LECs

**Figure 3:**
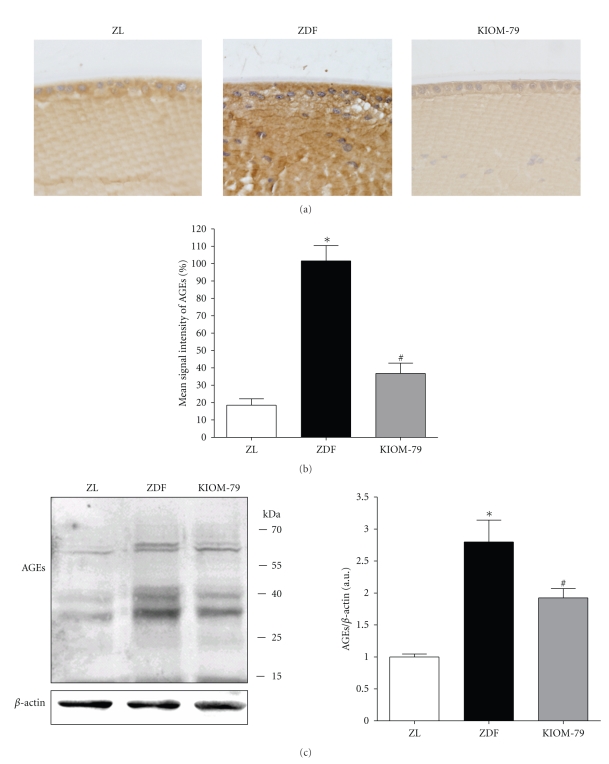
AGE accumulation. (A) Immunohistochemical localization of AGEs in lens from a normal Zucker lean rat (ZL), vehicle-treated ZDF rat (ZDF) and ZDF rat treated with KIOM-79 (KIOM-79). A strong AGE immunoreactivity was observed in the cytoplasm of LECs and lens fibers of vehicle-treated ZDF rats. In contrast, immunoreactivity in lenses of KIOM-79-treated ZDF rats was decreased. X400 magnification. (B) Quantitative analysis of AGE immunoreactive intensity. (C) Western blot analysis of AGEs. Values in the bar graphs represent means ± SE, *n* = 8. *P < .01 versus normal ZL rats, ^#^ *P* < .01 versus vehicle-treated ZDF rats.

We next examined the accumulation of AGEs in LECs by immunohistochemistry and western blot analysis. As shown in Figures 3(a) and 3(b), a striking increase in the immunoreactivity of AGEs was observed in cataractous lenses of vehicle-treated ZDF rats. Compared with normal ZL rats, this staining was observed in the cytoplasm of LECs and in the deeper regions of cortical fibers. By western blotting analysis, the multiple and intensive bands were detected in cataractous lenses of the ZDF rats. The expression of AGEs was 2.7-fold higher in the ZDF rat than that in the normal ZL rats (Figure 3(c)). However, the treatment of KIOM-79 reduced the AGEs deposited in LECs and cortical fibers.

### 3.6. Expression of iNOS in LECs

**Figure 4:**
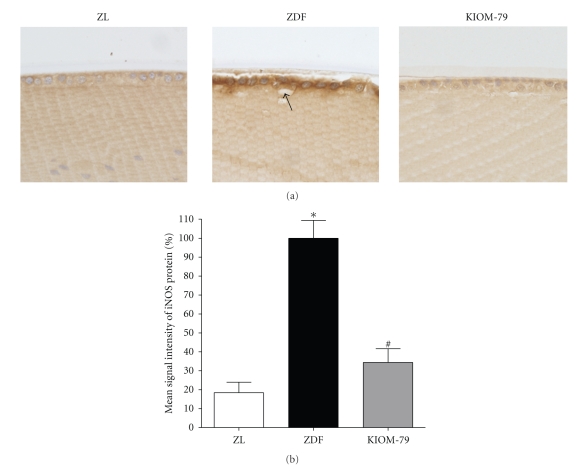
The expression pattern of iNOS. (a) Immunohistochemical localization of iNOS protein. iNOS immunoreactivity (arrow) was observed in the cytoplasm of LECs of diabetic lenses. The immunoreactivity in KIOM-79-treated rats was decreased in its intensity. ZL: normal Zucker lean rat; ZDF: vehicle-treated ZDF rat; KIOM-79: ZDF rat treated with KIOM-79. X400 magnification. (b) Quantitative analysis of iNOS protein signal intensity. Values in the bar graphs represent means ± SE, *n* = 8. *P < .01 versus normal ZL rats, ^#^ *P* < .01 versus vehicle-treated ZDF rats.

The localization of iNOS protein was detected by immunohistochemistry. In cataractous lenses of vehicle-treated ZDF rats, iNOS was markedly expressed in the cytoplasm of LECs compared with normal ZDF rat lenses. However, a remarkable reduction in the immunoreactivity of iNOS was observed in LECs of KIOM-79-treated rats (Figure 4(a)). In quantitative analysis, the intensity of iNOS was increased fivefold in vehicle-treated ZDF rats compared to normal ZL rats. This change was reduced by 63% with KIOM-79 treatment (*P* < .01) (Figure 4(b)).

### 3.7. Activation of NF-*κ*B in LECs

**Figure 5:**
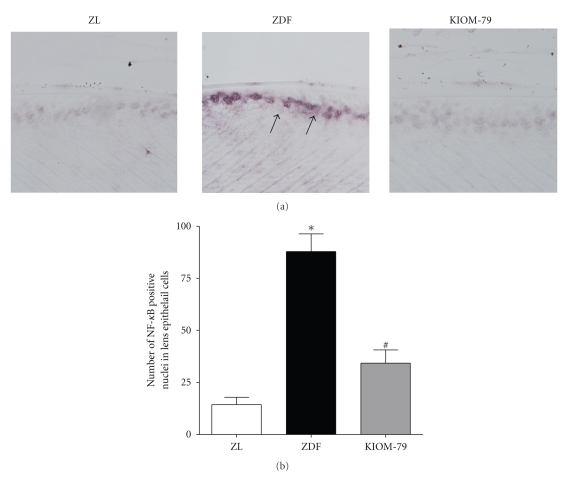
Distribution of NF-*κ*B in LECs detected by southwestern histochemistry. (a) Representative photomicrograph of lens from a normal Zucker lean rat (ZL), vehicle-treated ZDF rat (ZDF), and ZDF rat treated with KIOM-79 (KIOM-79). Positive signals (arrow) for activated NF-*κ*B were mainly detected in nuclei of LECs of the vehicle-treated ZDF rat. X400 magnification. (b) Quantitative analysis of positive cells in LECs. Values in the bar graphs represent means ± SE, *n* = 8. *P < .01 versus normal ZL rats, ^#^ *P* < .01 versus vehicle-treated ZDF rats.

NF-*κ*B activity was detected by southwestern histochemistry. This technique allows the localization of the activated nuclear factor in the cellular nucleus. Using this southwestern histochemistry method, we observed that a marked NF-*κ*B activity was mainly found in the LEC nuclei of vehicle-treated ZDF rats (Figure 5(a)). However, in the normal ZL rats, the signals of activated NF-*κ*B were rarely detected. Moreover, morphometric analysis showed that the expression of activated NF-*κ*B in vehicle-treated ZDF rats was 6.0-fold higher than normal ZL rats whereas KIOM-79 significantly inhibited the expression of activated NF-*κ*B by 60%, when compared to vehicle-treated ZDF rats (Figure 5(b), *P* < .01).

## 4. Discussion

Apoptosis of LECs induced by ultraviolet radiation, oxidative stress and hyperglycemia is associated with cataractogenesis [3, 4]. In addition, the inhibition of LEC apoptosis has been shown to reduce the formation of cataract [4, 31]. Therefore, identification of a treatment which blocks LEC apoptosis and prevents cataract formation would present a potential therapeutic strategy for diabetic cataract. 

In this study, to determine the preventive effect of KIOM-79 on LEC apoptosis, ZDF rats were treated with KIOM-79 for 13 weeks. The ZDF rat is one of the attractive models for type 2 diabetes based on its impaired glucose tolerance caused by an inherited insulin-resistance gene. The ZDF rats exhibited hyperglycemia and diabetic cataract at 21 weeks old, which is in agreement with previous reports [32]. In our previous study, serum AGE levels were significantly increased, and AGEs were highly accumulated in the glomeruli and tubulointerstitium of 20-week-old ZDF rats [33]. In ZDF rats at 21 weeks of age, AGEs were accumulated in LECs of cataractous lenses. These LECs with increased AGEs underwent apoptosis under diabetic conditions. Moreover, both the expression of iNOS and activation of NF-*κ*B in LECs were markedly increased during cataract development. Interestingly, oral administration of KIOM-79, a mixture of the 80% ethanol extract of parched Puerariae radix, gingered Magnoliae cortex, Glycyrrhizae radix, and Euphorbiae radix, prevented the enhanced apoptosis, AGE accumulation, iNOS expression, and NF-*κ*B activation in LECs. 

We identified four major compounds (magnolol, honokiol, glycyrrhizin, and puerarin) in KIOM-79. Each component is considered to have antidiabetic effects [29, 34–36]. Furthermore, it was previously reported that puerarin confers a protective effect against apoptosis in diabetic rat LECs [37]. Magnolol prevents oxidized low density lipoprotein-(oxLDL-) induced vascular endothelial apoptosis. Honokiol suppresses NF-*κ*B activation, and NF-*κ*B-regulated gene expression through the inhibition of IKKs [38] and attenuates oxLDL-induced apoptosis in vascular endothelial cells [39]. Glycyrrhizin inhibits 3-morpholinosydnonime-induced apoptosis in lung epithelial cells [40]. Therefore, the anti-apoptotic activity of KIOM-79 against diabetes-induced LEC apoptosis may be considered to be due to the effects of these compounds. 

NO is synthesized from L-arginine by NO synthase. Inducible NO synthase (iNOS), one of the three isoforms of NOS, is induced by various cytokines and AGEs [14, 15]. The induction of iNOS results in sustained and upregulated release of excessive amounts of NO, which is cytotoxic to neighboring cells [16, 17]. NO is also known to influence apoptosis in a variety of models [41]. In several ocular diseases, such as uveitis, retinitis, and glaucoma, the alteration of iNOS and abnormal production of NO were observed [18, 19]. The levels of NO in the vitreous body were increased in diabetic retinopathy and traumatic cataract [42, 43]. The apoptosis of human LECs stimulated by lipopolysaccharide is accompanied by iNOS induction [44]. Moreover, an iNOS inhibitor attenuates cataract formation *in vitro*. In contrast, an NO generator accelerates cataract formation [22]. These findings support the hypothesis that iNOS plays a key role in controlling LEC function and apoptosis. 

NF-*κ*B is a multiprotein complex that can activate many kinds of genes involved in cellular functions. In unstimulated cells, NF-*κ*B resides in the cytoplasm. Pathogenic stimuli allow NF-*κ*B to enter the nucleus where NF-*κ*B binds to DNA recognition sites in regulatory regions of target genes [45–47]. The interaction of AGEs and their receptors (RAGE) leads to NF-*κ*B activation [48]. NF-*κ*B activation stimulated by the AGE/RAGE interaction contributes to the development of diabetic ocular complications, such as diabetic retinopathy and lacrimal gland dysfunction [12, 49–51]. AGEs induce a dose-dependent activation of NF-*κ*B in LECs *in vitro* [9]. Moreover, NF-*κ*B can induce the expression of iNOS mRNA through NF-*κ*B binding sites in the iNOS promoter [52–56]. 

At present, KIOM-79 induced only a minor decrease of blood glucose levels and had no effect on body weight or HbA1c. However, KIOM-79 prevented lens opacity and significantly inhibited apoptosis, AGE accumulation, NF-*κ*B activation, and iNOS induction in LECs when compared with vehicle-treated ZDF rats. Thus, these results strongly suggest that the anticataract effect of KIOM-79 is unrelated to lowering blood glucose but it is due to the inhibition of LEC apoptosis and AGE accumulation in the lenses of ZDF rats. Our previous studies also showed that KIOM-79 had an inhibitory effect of AGE formation *in vitro* and reduced AGE accumulation in the kidneys of STZ-induced diabetic rats, as well as, in the retinas of db/db mice [24, 27]. KIOM-79 has also been shown to prevent apoptosis of pancreatic betacells through the inhibition of the generation of reactive oxygen species [26]. In addition, KIOM-79 reduces the production of nitrite in lipopolysaccharide-stimulated murine macrophages [23]. 

Taken together, our results demonstrated a preventive effect of KIOM-79 on the formation of diabetic cataract observed in ZDF rats. This finding suggests that the mechanism of KIOM-79 may be associated, in part, with the inhibition of AGE accumulation. However, lens opacification is a complex phenomenon. Glycation represents only one of the contributory factors in lens opacification. Other factors, such as the polyol pathway and oxidative stress have also been implicated in the development of diabetic cataract [1]. Therefore, it remains to be clarified whether KIOM-79 also provides an aldose reductase inhibiting activity or antioxidant action.

**Figure 6:**
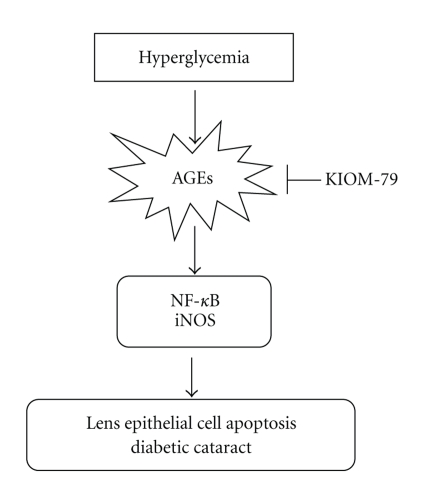
Proposed mechanism for the protective effect of KIOM-79 against lens epithelial cell apoptosis in Zucker diabetic fatty rats.

 In summary, KIOM-79 successfully prevented lens opacity in ZDF rats. KIOM-79 also had an anti-apoptotic effect on LECs via the suppression of AGE accumulation and its related signals including NF-*κ*B activation, and iNOS expression (Figure 6). Taken together, these results indicate that treatment with KIOM-79 could be a valuable therapeutic tool for diabetic cataract.

## Figures and Tables

**Table 1:** Amounts of puerarin, glycyrrhizin, honokiol, and magnolol in KIOM-79.

Compound	Content (mean ± SD, *n* = 3)
	%
Puerarin	4.82 ± 0.05
Glycyrrhizin	2.17 ± 0.04
Honokiol	0.87 ± 0.05
Magnolol	1.69 ± 0.10

**Table 2:** Metabolic and physical parameters.

	ZL	ZDF	KIOM-79
Body weight (g)	338.5 ± 40.5	433.2 ± 69.4*	414.6 ± 45.0
Blood glucose (mg/dl)	92.9 ± 10.8	489.8 ± 38.0*	391.7 ± 113.2^†^
HbA1c (%)	3.7 ± 0.1	7.88 ± 1.3*	7.1 ± 0.6

ZL: normal Zucker lean rats, ZDF: vehicle-treated Zucker diabetic fatty rats, and KIOM-79: Zucker diabetic fatty rats treated with KIOM-79 (50 mg/kg body weight). All data were expressed as mean ± SD. *P < .01 versus normal ZL rats, ^†^ *P* < .05 versus untreated ZDF rats.
